# Painting chromosomes in the nucleus

**DOI:** 10.7554/eLife.47468

**Published:** 2019-05-14

**Authors:** Cori K Cahoon, Diana E Libuda

**Affiliations:** 1 Department of BiologyUniversity of OregonEugeneUnited States; 2 Institute of Molecular BiologyUniversity of OregonEugeneUnited States

**Keywords:** DNA FISH, genome architecture, oligopaint, *C. elegans*

## Abstract

A multiplexed approach to DNA FISH experiments has been used to visualize the three-dimensional organization of chromosomes and specific chromosomal regions in *C. elegans.*

**Related research article** Fields BD, Nguyen SC, Nir G, Kennedy S. 2019. A multiplexed DNA FISH strategy for assessing genome architecture in *C. elegans*. *eLife* **8**:e42823. doi: 10.7554/eLife.42823

The genome of a mammal consists of about two meters of DNA and this DNA must somehow fit inside a nucleus that has a diameter of just ~10 microns (Misteli, 2008). To achieve this feat, the DNA interacts with various proteins to form a very compact and highly ordered complex called chromatin. The genetic material inside the nucleus is also divided into a number of chromosomes.

Recent advances in molecular biology and imaging techniques have begun to shed light on the organization of the DNA, chromatin, and chromosomes within the nucleus. The transcriptional profile of a gene directly influences its position within the nucleus: more active genes are localized near nuclear pores, whereas less active genes are positioned away from these pores (Casolari et al., 2004; Capelson et al., 2010; Pascual-Garcia and Capelson, 2014). It is also known that chromatin is arranged in loops, and that individual chromosomes are separated into non-overlapping regions called chromosome territories (Rao et al., 2014; Wang et al., 2016). However, there are many aspects of the organization of DNA, chromatin, and chromosomes within the nucleus that are not fully understood.

A technique called DNA FISH (short for fluorescence *in situ* hybridization) has made crucial contributions to our current understanding of chromatin structure, and the introduction of 'oligopaint' technology has made this approach cheaper, faster and more adaptable (Beliveau et al., 2012). Similar to any oligo, an oligopaint is essentially a length of synthetic DNA that has been designed to bind or hybridize to both natural DNA and to other synthetic DNA molecules. Now, in eLife, Scott Kennedy of Harvard Medical School and colleagues – including Brandon Fields (who is also at the University of Wisconsin-Madison) and Son Nguyen as joint first authors, and Guy Nir – report how they have developed a multiplexed version of the oligopaint technology that allows them to simultaneously visualize all six of the chromosomes in the worm *Caenorhabditis elegans* at virtually any developmental time and in any cell type (Fields et al., 2019).

Fields et al. used a combination of oligopaints, bridging oligos and detection oligos to study genome organization in *C. elegans*. Each of the 170,594 oligopaints in the library was 150 bases long and contained four 'barcodes' plus a 42-base region that hybridized to a specific region on a specific chromosome in *C. elegans* (Figure 1). Each bridging oligo contained a region that hybridized to one of the barcodes on the oligopaint, and a region that hybridized to a detection oligo. The detection oligo was fused to a fluorescent dye, which made it possible to see where the oligopaint was, and hence where the specific region of the chromosome was. Fields et al. used three different fluorescent dyes (red, green, and blue) to label the oligopaints in the experiments. Additionally, pairwise combinations of these fluorescent dyes can be employed on a single oligopaint by using two barcodes with two bridging oligos and two detection oligos, which generates a total of six distinct fluorescent patterns.

**Figure 1.**
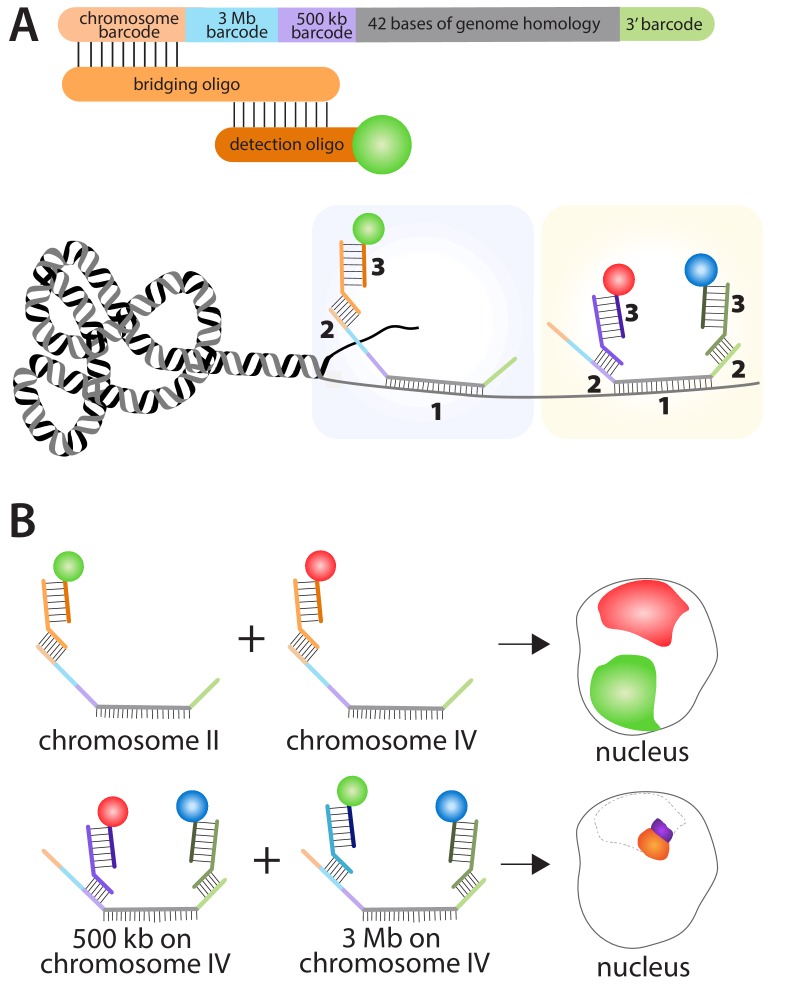
Using the multiplexed DNA FISH method to visualize chromosomes in *C. elegans.* (**A**) The oligopaints used by Fields et al. contained a chromosome barcode (orange), a 3 Mb barcode (blue), a 500 kb barcode (purple), a 42-base region (grey) that hybridizes to a region of interest on a specific chromosome, and a 3′ barcode (green). The first stage in the process (pale blue boxed region) involves the hybridization of the 42-base region in the oligopaint to its target (1); a bridging oligo then hybridizes to one of the barcodes on the oligopaint (2); a detection oligo with a fluorescent dye then hybridizes to the bridging oligo (3). It is also possible (yellow boxed region) for a second bridging oligo to hybridize to the 3′ barcode on the oligopaint and increase the number of genome regions that can be detected within a single nucleus. (**B**) This approach can be used to determine the location of different chromosomes within the nucleus by using bridging oligos that bind to the chromosome barcode on the oligopaints specific to each chromosome, along with detection oligos with different fluorescent dyes (top schematic: green for chromosome II; red for chromosome IV). Likewise, the location of different regions within the same chromosome can be determined by using bridging oligos that bind to either the 3 Mb or 500 kb barcodes on the oligopaint specific to one chromosome (bottom schematic). Here blue fluorescence from the 3′ barcode and red fluorescence from the 500 kb barcode combine to give purple fluorescence; blue fluorescence from the 3′﻿ barcode and green fluorescence from the 3 Mb barcode combine to give orange fluorescence. The dotted line represents the region of the nucleus occupied by chromosome IV.

The barcodes make it possible to label (and subsequently visualize) either a whole chromosome, a 3 megabase subregion of a chromosome, or a 500 kilobase subregion (Figure 1). These barcode sequences also enable the re-amplification of the oligopaint library to create an infinitely renewable library. Moreover, the fluorescently pre-labeled detection oligos eliminate the need to directly label each oligopaint, which when combined with the bridging oligos, makes the oligopaint method both very cost effective and versatile. Indeed, Fields et al. were able to visualize all six chromosomes within multiple tissues types in *C. elegans* including oocytes, germ line cells, intestinal cells, hypodermal cells, and neuronal cells.

Highlighting the power of this method, Fields et al. used the multiplexed DNA FISH method to explore important questions about genome organization. For example, does aging alter the organization of the genome in *C. elegans*? Remarkably, they observed that the intestinal cells in one-day-old animals displayed distinct chromosome territories that were not present in ten-day-old animals. The disorganization of higher-order chromosome structures has been implicated in aging-related diseases (Evans et al., 2019), but further studies are needed to understand the mechanistic relationship between aging and genome organization. Fields et al. were also able to identify genes that were involved in establishing and/or maintaining chromosome territories (such as the gene that encodes a protein called MES-3).

The ability of multiplexed DNA FISH to visualize chromosomes in intact animals will help the *C. elegans* community to address fundamental questions about the establishment, maintenance and regulation of higher-order chromatin organization in this important model organism. Further, combining this method with cell lineage maps for *C. elegans* could make it possible to understand the effect of development and cell differentiation on genome organization. Indeed, oligopaints give the *C. elegans* community the opportunity to illuminate how the organization and regulation of the genome are shaped by multiple processes and cell types within a developing and aging organism.
